# Delayed Surgery, Medial Meniscus Injury, and Younger Age Are All Factors Significantly Associated With High-Grade Knee Laxity Before ACL Reconstruction

**DOI:** 10.1177/23259671251356698

**Published:** 2025-07-25

**Authors:** Riccardo Cristiani, Eric Hamrin Senorski, Camilo P. Helito, Kristian Samuelsson, Karl Eriksson, Anders Stålman

**Affiliations:** † Department of Molecular Medicine and Surgery, Section of Sports Medicine, Karolinska Institutet, Stockholm, Sweden; ‡ Stockholm Sports Trauma Research Center, FIFA Medical Centre of Excellence, Sophiahemmet, Stockholm, Sweden; § Department of Health and Rehabilitation, Institute of Neuroscience and Physiology, Sahlgrenska Academy, University of Gothenburg, Gothenburg, Sweden; ‖ Hospital Sírio Libanês, São Paulo, São Paulo, Brazil; ¶ Grupo de Joelho, Instituto de Ortopedia e Traumatologia, Hospital das Clínicas HCFMUSP, Faculdade de Medicina da Universidade de São Paulo, São Paulo, São Paulo, Brazil; # Department of Orthopaedics, Institute of Clinical Sciences, The Sahlgrenska Academy, University of Gothenburg, Gothenburg, Sweden; ** Department of Orthopaedics, Stockholm South Hospital, Stockholm, Sweden; †† Karolinska Institutet, Stockholm, Sweden; Investigation performed at Capio Artro Clinic & Stockholm Sports Trauma Research Center, Karolinska Institutet, Stockholm, Sweden

**Keywords:** anterior cruciate ligament, ACL, meniscus, knee laxity, delayed surgery, arthrometer

## Abstract

**Background:**

The influence of patient demographics, associated meniscal injuries, and especially the time from injury to surgery on knee laxity before anterior cruciate ligament reconstruction (ACLR) remains insufficiently explored.

**Purpose:**

To identify factors associated with high-grade knee laxity before ACLR.

**Study Design:**

Case-control study; Level of evidence, 3.

**Methods:**

Patients without concomitant ligament injuries who underwent primary ACLR at Capio Artro Clinic, Stockholm, Sweden, from January 2000 to December 2023 were identified. Demographic data (age and sex), concomitant medial or lateral meniscus injury, time from injury to surgery, and instrumented knee laxity were reviewed. The KT-1000 arthrometer (134 N) was used to evaluate anterior knee laxity preoperatively. High-grade laxity was defined as a side-to-side (STS) difference >5 mm (International Knee Documentation Committee grade C or D). A logistic regression analysis was used to evaluate whether age at surgery, sex, medial or lateral meniscus injury, and time from injury to ACLR were associated with high-grade preoperative knee laxity.

**Results:**

A total of 7759 patients (53.9% male) were included, of whom 1596 had high-grade knee laxity (STS difference >5 mm) preoperatively. Younger age (≤26 years) (OR, 1.19; 95% CI, 1.06-1.33; *P* = .002), medial meniscus injury (OR, 1.44; 95% CI, 1.28-1.63; *P* < .001), and delayed surgery (6-12 months: OR, 1.23; 95% CI, 1.03-1.46; *P* = .02; 12-24 months: OR, 1.58; 95% CI, 1.29-1.92; *P* < .001; 24-48 months: OR, 1.86; 95% CI, 1.47-2.35; *P* < .001; >48 months: OR, 2.23; 95% CI, 1.77-2.82; *P* < .001) increased the odds of high-grade preoperative knee laxity. Female sex and lateral meniscus injury were not associated with high-grade preoperative knee laxity.

**Conclusion:**

Age ≤26 years, medial meniscus injury, and delayed surgery (>6 months) were associated with high-grade knee laxity (STS difference >5 mm) before ACLR. To reduce the likelihood of knee laxity deterioration, ACLR should be performed within 6 months of the injury.

An anterior cruciate ligament (ACL) tear leads to greater anterior translation and internal rotation of the tibia.^3,19^ In addition to ACL injury, various factors can influence the degree of anterior tibial translation. The KT-1000 arthrometer (MEDmetric) is the most commonly used tool for measuring anterior tibial translation in ACL-injured knees.^4,5,21^ The degree of laxity measured during arthrometric evaluation may affect treatment decisions and prognosis.

Notably, previous studies have demonstrated that preoperative knee laxity is the most important determinant of postoperative laxity after ACL reconstruction (ACLR).^4^ A side-to-side (STS) laxity difference >5 mm, classified as abnormal by the International Knee Documentation Committee (IKDC) form,^13^ has been identified as the most critical risk factor for maintaining such laxity after ACLR.^4^ Abnormal postoperative knee laxity might affect ACL graft remodeling^25^ and has been associated with an increased risk of revision ACLR.^5,10^ Cristiani et al^5^ found that high-grade knee laxity (STS difference >5 mm) 6 months postoperatively resulted in a 2.26-fold higher risk of revision ACLR within 5 years, and Fiil et al^10^ demonstrated a 5.51-fold increased risk of revision within 2 years for patients with an STS difference >5 mm at 1 year postoperatively. In addition, persistent laxity may increase shear forces across the cartilage, leading to joint surface degeneration and a higher risk of osteoarthritis.^33^ These findings emphasize the importance of understanding the factors that contribute to preoperative laxity, as persistent abnormal laxity after surgery can lead to significant clinical consequences. Gaining such insights can enhance preoperative evaluation and help identify patients at higher risk of poor surgical outcomes. Furthermore, if some of the factors affecting preoperative knee laxity are modifiable, there may be opportunities to improve postoperative outcomes. Nonetheless, the effect of patient demographics, associated meniscal injuries, and particularly the time from injury to surgery on preoperative knee laxity has not been thoroughly investigated.

The purpose of this study was to identify factors associated with high-grade knee laxity (STS difference >5 mm) before ACLR. It was hypothesized that a delay in ACLR, medial meniscus injury, female sex, and younger age would be associated with increased odds of high-grade preoperative knee laxity.

## Methods

### Participants

Ethical approval for this study was obtained by the regional ethics committee, Karolinska Institutet (Diarienumber 2016/1613-31/32). The STROBE (Strengthening the Reporting of Observational Studies in Epidemiology) statement was used as guidance to report this study to increase scientific quality.^34^

Patients without concomitant ligament injuries who underwent primary ACLR at Capio Artro Clinic, Stockholm, Sweden, from January 2000 to December 2023 were assessed for eligibility. All included patients had a complete data set available. Patients were excluded if they had a previous ACL injury or reconstruction.

### Arthrometric Evaluation

Preoperative instrumented knee laxity was evaluated at our outpatient clinic using the KT-1000 arthrometer. All measurements were performed by experienced sports physical therapists. A standard anterior tibial load of 30 pounds (134 N) was applied at 20° of knee flexion. At least 3 measurements were taken for each knee, and the median value was recorded. The STS difference in anterior tibial displacement between the ACL-injured knee and the uninjured contralateral knee (measured in millimeters) was documented. High-grade knee laxity was defined as an STS difference >5 mm (corresponding to IKDC grades C and D).^13^ All measurements were conducted either the day before or the day of surgery.

### Data Sources

Data were extracted from the Capio Artro Clinic database. The variables collected were age at surgery, sex, medial meniscus injury, lateral meniscus injury, time from injury to surgery and KT-1000 arthrometric measurements. Meniscal injuries were diagnosed at the time of surgery.

### Statistical Analysis

The Statistical Package for the Social Sciences (Version 25.0; IBM Corp) was used for the statistical analysis. All the variables were summarized with standard descriptive statistics such as mean, median, standard deviation, range, or frequency.

A multivariable logistic regression analysis was performed with age at surgery, sex, medial meniscus injury, lateral meniscus injury, and time from injury to surgery as independent variables and high-grade preoperative knee laxity (STS difference >5 mm) as the dependent variable. Age was dichotomized into unbiased classes close to the median (≤26 years vs >26 years). Time from injury to surgery was stratified in monthly intervals as follows: 0 to 3, 3 to 6, 6 to 12,12 to 24, 24 to 48, and >48 months. The 0- to 3-month group was selected as the reference group, with which the other time from injury to surgery groups were compared. This classification of time from injury to surgery was selected because these time intervals may serve as practical benchmarks for clinical decision-making. All the relationships were expressed as odds ratios with 95% confidence intervals. The level of significance in all analyses was 5% (2-tailed).

## Results

A total of 8110 patients were reviewed for eligibility. After excluding patients with previous ACL injury or reconstruction (n = 351), 7759 patients were included. Patient characteristics are summarized in Table 1.

**Table 1:** Patient Characteristics (N = 7759 patients)*^*a*^*

	Value	Preoperative STS Difference, mm
Age at surgery, y	28.2 ± 10.8 (range, 8-69)	
≤26	3858 (49.7); 19.2 ± 3.8	3.6 ± 2.6
>26	3901 (50.3); 37.1 ± 7.7	3.3 ± 2.8
Sex		
Female	3575 (46.1)	3.6 ± 2.7
Male	4184 (53.9)	3.5 ± 2.8
Medial meniscus injury	1941 (25.0)	4.0 ± 2.8
Lateral meniscus injury	1999 (25.8)	3.5 ± 2.7
Time from injury to surgery, mo	15.7 ± 29.7	
0-3	1498 (19.3)	3.2 ± 2.5
3-6	2155 (27.8)	3.3 ± 2.6
6-12	2012 (25.9)	3.5 ± 2.7
12-24	1001 (12.9)	3.9 ± 2.8
24-48	543 (7.0)	4.0 ± 3.0
>48	550 (7.1)	4.2 ± 3.1

a Data are reported as n (%) or mean ± SD unless otherwise indicated. STS, side-to-side.

A total of 1596 patients had high-grade knee laxity (STS difference >5 mm) preoperatively. Logistic regression analysis showed that high-grade preoperative knee laxity was associated with younger age (≤26 years) (OR, 1.19; 95% CI, 1.06-1.33; *P* = .002), medial meniscus injury (OR, 1.44; 95% CI, 1.28-1.63; *P* < .001), and delayed surgery (6-12 months: OR, 1.23; 95% CI, 1.03-1.46; *P* = .02; 12-24 months: OR, 1.58; 95% CI, 1.29-1.92; *P* < .001; 24-48 months: OR, 1.86; 95% CI, 1.47-2.35; *P* < .001; >48 months: OR, 2.23; 95% CI, 1.77-2.82; *P* < .001). There was no association between female sex or lateral meniscus injury and high-grade preoperative knee laxity (Table 2).

**Table 2:** Factors Associated With High-Grade Laxity (STS Difference >5 mm) Before ACLR in Logistic Regression Analysis*^*a*^*

Factor	Regression Coefficient (β)	SE	OR (95% CI)	*P* Value
Age at surgery ≤26 y	0.33	0.06	1.19 (1.06-1.33)	**.002**
Female sex	0.008	0.05	1.00 (0.90-1.13)	.89
Medial meniscus injury	0.37	0.06	1.44 (1.28-1.63)	**<.001**
Lateral meniscus injury	0.37	0.07	1.07 (0.94-1.22)	.28
Time from injury to surgery, mo				
0-3 (reference group)				
3-6	0.03	0.09	1.03 (0.86-1.23)	.73
6-12	0.20	0.09	1.23 (1.03-1.46)	**.02**
12-24	0.45	0.10	1.58 (1.29-1.92)	**<.001**
24-48	0.62	0.12	1.86 (1.47-2.35)	**<.001**
>48	0.80	0.12	2.23 (1.77-2.82)	**<.001**

a Bold *P* values indicate statistical significance. ACLR, anterior cruciate ligament reconstruction; SE, standard error; STS, side-to-side.

## Discussion

The most important finding of the present study was that delayed ACLR, medial meniscus injury, and younger age were associated with high-grade (STS difference >5 mm) pre-reconstruction laxity.

The effect of time from injury to surgery on preoperative knee laxity has not been thoroughly investigated. The key finding of this study was that delaying ACLR for >6 months was associated with an increased likelihood of high-grade preoperative knee laxity. Interestingly, the odds of having high-grade preoperative knee laxity progressively increased with time, with the group undergoing ACLR >48 months after the injury showing the highest odds. This phenomenon may be attributed to the biomechanical consequences of prolonged ACL deficiency. When the ACL is ruptured, the primary restraint against anterior tibial translation is lost. This situation forces secondary stabilizers—such as the joint capsule, anterolateral structures and Kaplan fibers, collateral ligaments, and other periarticular structures—to compensate for the increased knee laxity.^30^ Over time, repeated loading and stress on these structures during activities of daily living and/or sports may cause progressive stretching of these secondary stabilizers.^6^ This further increases knee laxity, which cannot be compensated for with a future ACLR. This hypothesis is also supported by the study of Magnussen et al,^23^ who found that delaying ACLR for >6 months was associated with more than double the odds of testing positive for high-grade Lachman, pivot-shift, and anterior drawer tests, compared with early (<3 months) surgery. Similarly, Signorelli et al^32^ examined the effect of the time between ACL injury and surgery on preoperative knee laxity and reported that longer delays resulted in greater knee laxity, particularly in anteroposterior displacement at 90° of flexion and varus-valgus rotation at 30°. These findings underscore the importance of timely surgical intervention in patients with ACL injuries to reduce progressive laxity and potentially improve postoperative outcomes. To our knowledge, this is the first study to analyze the effect of surgical delay on preoperative knee laxity using objective measurements with the KT-1000 arthrometer.

There has been limited research on the effect of patient age on pre-reconstruction knee laxity; however, previous studies have reported greater knee laxity after ACLR in younger patients. Marchand et al^24^ observed increased anterior tibial translation in patients <20 years of age at a mean follow-up of 26 months after ACLR. Cristiani et al^4^ found that age <30 years was a risk factor for abnormal knee laxity (STS difference >5 mm) after ACLR. Similarly, we found age ≤26 years to be a factor associated with high-grade (STS difference >5 mm) pre-reconstruction laxity. This implies that younger patients may have increased knee laxity postoperatively, not only because of possible graft elongation,^24^ but also because of their increased knee laxity preoperatively.^4^ It has been suggested that older patients may have increased knee “stiffness” due to the progressive degenerative joint changes.^4^

The effect of patient sex on knee laxity has primarily been studied after ACLR, whereas its influence before ACLR remains less explored. While some authors have reported increased knee laxity in females after ACLR,^12,29,31^ others^4,8,18^ have not found any relationship between patient sex and postoperative arthrometric evaluation. Similarly, we were unable to demonstrate any increased odds of high-grade laxity in females within this large cohort of patients.

As hypothesized, the presence of a medial meniscus injury increased the odds of high-grade preoperative knee laxity. Previous research has emphasized the critical role of the medial meniscus as a secondary stabilizer, particularly in controlling anterior tibial translation in ACL-deficient knees.^1,6,11,35^ Meniscal repair during ACLR has been shown to aid in restoring knee laxity,^4,7^ reduce the load on the ACL graft, and ultimately decrease the risk of graft failure.^27,28^ On the other hand, the presence of a lateral meniscus injury did not increase the odds of having high-grade preoperative knee laxity. Similar results have been reported in previous studies,^6,26^ reinforcing the consistency of these findings across research. Unlike the medial meniscus, the lateral meniscus is less securely attached to the tibial plateau, has no attachments to the lateral collateral ligament, and lacks a posterior “wedge effect” to resist anterior tibial translation relative to the femur.^20^ However, there is evidence that the lateral meniscus plays a significant role in controlling rotational laxity.^17,26^

High-grade preoperative knee laxity may have negative consequences for patients with ACL injuries. Magnussen et al^22^ reported that high-grade preoperative laxity, identified through manual examination under anesthesia, is linked to a significantly higher likelihood of revision ACLR within 2 years from the primary surgery. Moreover, an STS difference >5 mm (KT-1000 arthrometer) before ACLR has been identified as the most significant risk factor for persistent high-grade knee laxity (STS difference >5 mm) even after surgery.^4^ Fiil et al^10^ and Cristiani et al^5^ have reported that an STS laxity >5 mm after ACLR was associated with an increased risk of revision ACLR within 2 and 5 years of surgery, respectively. Persistent knee laxity after ACLR can also increase stress on joint surfaces and the menisci, increasing the risk of subsequent meniscal and cartilage injuries, which over time may contribute to the development of osteoarthritis.^14,33^ A relationship between KT-1000 measurements and the degree of osteoarthritis has been reported.^33^ Finally, persistent high-grade knee laxity may also affect the proper remodeling of the ACL graft, potentially compromising its long-term function.^25^ This study suggests that the advantages of early ACLR include not only a reduced risk of associated meniscal and cartilage injuries as reported by other studies,^2,6,9^ but also a lower likelihood of altered knee kinematics and increased knee laxity. When ACLR is indicated, performing the procedure within 6 months of injury may reduce the risk of increased knee laxity. In cases of delayed ACLR and increased preoperative knee laxity, adding a lateral extra-articular procedure may help reduce postoperative laxity.^15,16^

There are several strengths in this study. First, the cohort included was large (7759 patients) and diverse in terms of age and time between injury and ACLR. These characteristics allowed a robust logistic regression analysis and made the results generalizable. Second, knee laxity was measured with the KT-1000 arthrometer, which offers greater precision than clinical tests by providing a quantitative assessment of anterior tibial translation. Third, all arthrometric measurements were performed at the same institution with the same technique and anterior tibial displacement force. Finally, we evaluated the association between several time from injury to surgery intervals and preoperative knee laxity.

This study also has several limitations. First, it is commonly noted that arthrometric evaluations may be influenced by interobserver variability, and measurements obtained from awake patients may differ from those conducted under anesthesia due to muscle guarding. However, the study included a large sample, and all the physical therapists who conducted the measurements were experienced in using the KT-1000 arthrometer. Additionally, a standardized technique was used (knee at 20° of flexion and a standard anterior tibial translation force of 134 N), which helped minimize the effect of this potential limitation. Second, the extent and location of meniscal injuries were not characterized, as our database does not include this information. It was therefore not possible to assess the effect of different meniscal injury types or locations on preoperative knee laxity. Third, we had no data on other factors that may affect preoperative knee laxity, such as injury to the anterolateral structures of the knee or generalized joint laxity. Fourth, we have not studied the factors associated with high-grade preoperative rotatory laxity as the pivot-shift test is not documented in a standardized way in our database, making any evaluation challenging to conduct. Finally, the study included only patients who underwent surgical treatment and did not comprehensively explore the indications for surgery. However, in general, the primary indications for ACLR at our institution were patient-reported knee instability and/or a desire to return to pivoting sports.

## Conclusion

Younger age (≤26 years), medial meniscus injury, and delayed surgery (>6 months) were associated with high-grade (STS difference >5 mm) knee laxity before ACLR. To reduce the likelihood of knee laxity deterioration, ACLR should be performed within 6 months of the injury.
